# A Novel Ferroptosis-Related Signature for Prediction of Prognosis, Immune Profiles and Drug Sensitivity in Hepatocellular Carcinoma Patients

**DOI:** 10.3390/curroncol29100550

**Published:** 2022-09-27

**Authors:** Chuanbing Zhao, Zhengle Zhang, Jing Tao

**Affiliations:** Department of Pancreatic Surgery, Renmin Hospital of Wuhan University, Wuhan 430061, China

**Keywords:** hepatocellular carcinoma, novel ferroptosis-related gene signature, immunotherapy, prognosis, drug sensitivity

## Abstract

Hepatocellular carcinoma (HCC) is a malignant disease with an increasing incidence and a high mortality rate. Ferroptosis, a novel type of cell death, has been reported to be closely associated with the progression of HCC. The aim of our study was to construct a novel ferroptosis-related signature (nFRGs) for prediction of prognosis, immune features and drug sensitivity of HCC patients. Data were obtained from the TCGA, ICGC, GSE104580, CCLE and IMvigor210 datasets, and the least absolute shrinkage and selection operator (LASSO) was used to construct nFRGs. In addition, the analyses involved in prognoses, molecular function, stemness indices, somatic mutation, responses to immunologic therapy, efficacy of transcatheter arterial chemoembolization (TACE) therapy and drug sensitivity were performed using diverse packages of R 4.1.3 between the low- and high-risk groups. The nFRGs included seven ferroptosis-related genes. Our results showed that nFRGs was an independent risk factor for prognoses of HCC patients, and HCC patients in the high-risk group presented with worse prognosis. Compared with the results of other studies, nFRGs was superior to other promising signatures in predicting prognoses of patients with HCC. In addition, most of the enriched pathways of differentially expressed genes (DEGs) between these subgroups were related to immune features. The molecular functions, genetic mutation and mRNAsi were varied between the high- and low-risk groups. Moreover, we observed significant immunosuppression state in the high-risk group. Patients in the high-risk group might benefit from immunotherapy, whereas patients in the low-risk group may be susceptible to TACE therapy. Finally, five sensitive drugs and four sensitive drugs were screened for patients in the high- and low-risk groups, respectively. nFRGs may served as a novel biomarker of prognosis and aid in personalized therapeutic strategies for patients with HCC.

## 1. Introduction

In the latest global cancer statistics report published 2020, the incidence of HCC was 4.7% (ranking seventh among all cancers), with a mortality rate of 8.3% (ranking second among all cancers) [1]. Although innovative therapeutic strategies, including targeted therapy and immunotherapy, have made considerable progress in the treatment of HCC, the prognosis of HCC is still not satisfactory [2,3,4,5]. Thus, the identification of new prognostic markers and therapeutic targets to improve the outcome of HCC are among current research priorities.

Ferroptosis, distinct from cuproptosis and apoptosis, is a newly recognized regulated mode of cell death that results from iron-dependent lipid peroxidation and reactive oxygen species accumulation [6]. A growing body of evidence has indicated that ferroptosis-related pathways may inhibit the progression of a variety of tumors, such as colorectal cancer [7], head and neck cancer [8], and lung cancer [9]. In addition, metastasis, invasion, and drug resistance of HCC cells are regulated by ferroptosis-related pathways [10,11,12]. For example, the sensitivity of HCC cells to sorafenib and lenvatinib may be affected by regulatory mechanisms of ferroptosis [13,14]. More importantly, ferroptosis-related pathways may have an impact on the effectiveness of immunotherapy in patients with cancer [15]. Previous studies have demonstrated that ferroptosis exerts an impact on the immune response of immune cells [16,17]. Recent studies have indicated that ferroptosis is expected to become a promising novel therapeutic target for patients with tumors [18]. Moreover, a considerable number of ferroptosis-related genes have been identified. Although several previous studies have confirmed the clinical significance of ferroptosis-related genes in the treatment and prognosis of HCC [19,20,21,22], the critical regulators of ferroptosis in predicting prognoses, as well as immunotherapeutic response and drug sensitivity, are not yet clear.

In this study, we constructed and validated nFRGs for prediction of prognoses in patients with HCC. Moreover, we discuss the role of nFRGs in evaluating molecular function, somatic mutation and stemness index in HCC patients. Our results suggest that nFRGs achieved excellent results in predicting prognoses and played a key role in assessing immunotherapy response, TACE efficacy and drug sensitivity in HCC patients.

## 2. Materials and Methods

### 2.1. Data Collection

**Figure 1:**
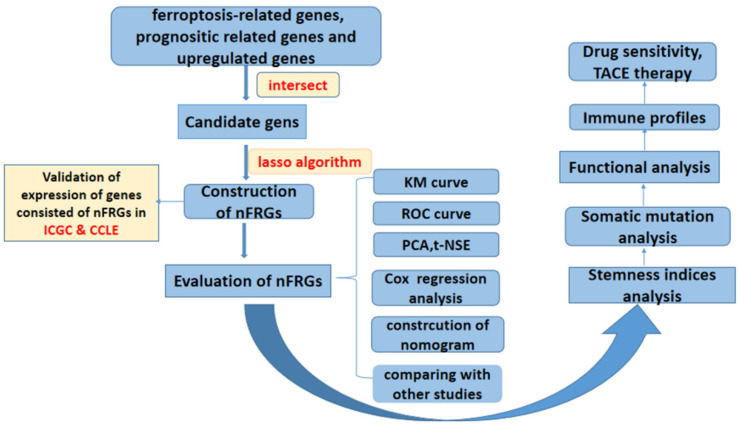
Flow chart of overall study design.

The RNA sequencing and corresponding clinical data of HCC patients were downloaded from the TCGA (https://cancergenome.nih.gov (accessed on 30 June 2022)), ICGC (https://dcc.icgc.org/ (accessed on 30 June 2022)) and GSE104580 databases (accessed on 30 June 2022). A list of ferroptosis-related genes was extracted from the FerrDb website (http://www.zhounan.org/ferrdb/ (accessed on 30 June 2022)) [23]. Furthermore, the mRNA expression matrix of HCC cell lines was available from the Cancer Cell Line Encyclopedia (CCLE) database (https://portals.broadinstitute.org/ccle (accessed on 2 July 2022)) [24]. Somatic mutation data for HCC patients were obtained from the Genomic Data Commons (GDC) database. In addition, we downloaded gene expression data and clinical information for patients in the IMvigor210 cohort using the “IMvigor210CoreBiologies” package [25]. The expression matrix data extracted from GSE104580 were normalized using the “limma” and “sva” packages. A flow chart of the study protocol is presented in Figure 1. 

### 2.2. Prognostic Analyses

#### 2.2.1. Construction and Validation of nFRGs 

The limma package was used to obtain differentially upregulated genes in the HCC tissues (logFc > 1.5, *p* < 0.05), and univariate Cox regression analysis was applied to screen for genes associated with overall survival (OS) (HR > 1.0, *p*.adj < 0.05). In addition, we obtained ferroptosis-related genes from the FerrDb database. These genes, which were taken from the above mentioned three groups, were intersected to obtain candidate genes. Moreover, the LASSO algorithm was implemented to select genes among the candidate genes for construction of nFRGs. In this study, risk scores were calculated as follows:

nFRGs risk score = Σni = 1Coefi × xi (xi: the expression level of each gene; Coefi: the variable coefficient calculated by the LASSO algorithm).

Based on the median risk scores, patients were assigned to low- and high-risk groups. The predictive performance of nFRGs was assessed by Kaplan–Meier curve (KM), receiver operating characteristic (ROC) curves, time ROC curve, principal component analysis (PCA) and t-distributed stochastic neighbor embedding (t-NSE) using the “survival”, “survminer”, “qROC”, “time ROC”, “STATS”, and “Rtsne” packages. nFRGs were externally validated using data from the ICGC database.

#### 2.2.2. Comparison of nFRGs with Other Gene Signatures

To evaluate the predictive performances of nFRGs for prognoses, we further compared nFRGs with the results of other studies using the “ggDCA” and “rms” packages to determine whether nFRGs were more informative in predicting overall survival (OS) in patients with HCC.

### 2.3. Molecular Function Analyses

The “limma” package was applied to screen differentially expressed genes (DEGs) between the high- and low-risk groups, and the “clusterProfiler” package was used to perform gene ontology (GO) and Kyoto Encyclopedia of Genes and Genomes (KEGG) analyses of DEGs. To identify potential distinctions in molecular mechanisms and biological functions between these subgroups, we further performed gene set enrichment analysis (GSEA) using the “clusterProfiler”, “enrichplot” and “circlize” packages based on the reference gene set (symbols.gmt v7.4.) in the MSigDB database (https://www.gsea-msigdb.org/gsea/ (accessed on 3 July 2022)).

### 2.4. Stemness Index Analyses

The one-class logistic regression (OCLR) algorithm was employed to calculate mRNAsi [26]. Based on this algorithm, we calculated mRNAsi for each sample. Moreover, we further determined whether mRNAsi varied between the low- and high-risk groups.

### 2.5. Somatic Mutation Analyses

As genetic mutations may likewise have an impact on the prognosis of HCC patients, we determined whether there were any differences in genetic mutations between these subgroups. We analyzed the top 20 mutated genes in the high-risk and low-risk groups and visualized the mutation details of these genes as waterfall plots using the “maftool” package. In addition, we calculated the tumor mutation burden (TMB) for each sample. Further comparisons of TMB for these subgroups were performed. Moreover, the impact of TMB on prognosis of HCC was explored using the “survival” package.

### 2.6. Immune Features

We quantified the infiltrating immune cells and immune function of each sample using the “GSVA” package and the “CIBERSORT” algorithm, and the differences in infiltration of immune cells and immune function between the high- and low-risk groups were determined. The tumor microenvironment (TME) was evaluated using the “estimate” package. Moreover, we determined whether the expression of common immune checkpoints differed in these subgroups using “limma” packages.

The “TIDE” algorithm was used to calculate the TIDE score for each sample [27]. Based on the TIDE score, we could predict the response to immunotherapy in patients with HCC. Then, we identified whether TIDE scores differed between the high-risk and low-risk groups, and we applied the TCIA database to identify whether there were any differences in response to PD-1 or CTLA-4 treatment between the high- and low-risk groups of patients with HCC [28].

It has been suggested that the T-cell inflammation score (TIS) can be evaluated to determine the efficacy of immunotherapy [29]. Accordingly, we calculated the TIS for each sample using the “limma” package and further assessed whether TIS varied in these subgroups. Recent studies have shown that CD8A and STAT1 appear to be highly expressed in responders to immune checkpoint inhibitors [30].

Furthermore, we calculated risk scores for each sample in the IMvigor210 cohort to validate the performance of nFRGs in assessing the response to immunotherapy.

### 2.7. The Role of nFRGs in Predicting Efficacy of TACE

TACE therapy is considered an option for patients with unresectable HCC. Hence, it is clinically significant to study the role of risk scores in assessing the efficacy of TACE. We calculated risk scores for each sample in GSE104580 and evaluated the performance of risk scores in predicting TACE responses.

### 2.8. The Role of nFRGs in Predicting Drug Sensitivity

The “pRRophetic” package was employed to select potentially sensitive drugs from more than 300 agents for high- and low-risk groups of HCC patients, with sensitivity indicators expressed as IC_50_ values.

## 3. Statistical Analyses

The overall survival (OS), disease-free survival (DSS), progression-free interval (PFI) and disease-free interval (DFI) of these subgroups were compared by using Kaplan–Meier method. In addition, we explored the relation between risk scores and clinical indicators (TNM-stage, T-stage, grade, vascular invasion, etc.) with the “complexHeatmap” and “limma” packages. Furthermore, a nomogram was created for prediction of OS in patients with HCC, and the nomogram was assessed with a calibration curve, KM method, PCA plot, and decision curve analysis (DCA) in the TCGA cohort using the “rms”, “survival”, “scatterplot3d” and “ggDCA” packages. In this study, statistical analyses were performed using R 4.1.3 (Creator: RickBecker, JohnChambers, AllanWilks. Location: New Zealand) and GraphPad Prism 8.0 (Creator: GraphPad Software).

## 4. Results

### 4.1. Excellent Predictive Performances for the Prognoses of Patients with HCC

#### 4.1.1. Construction and Validation of nFRGs

**Figure 2:**
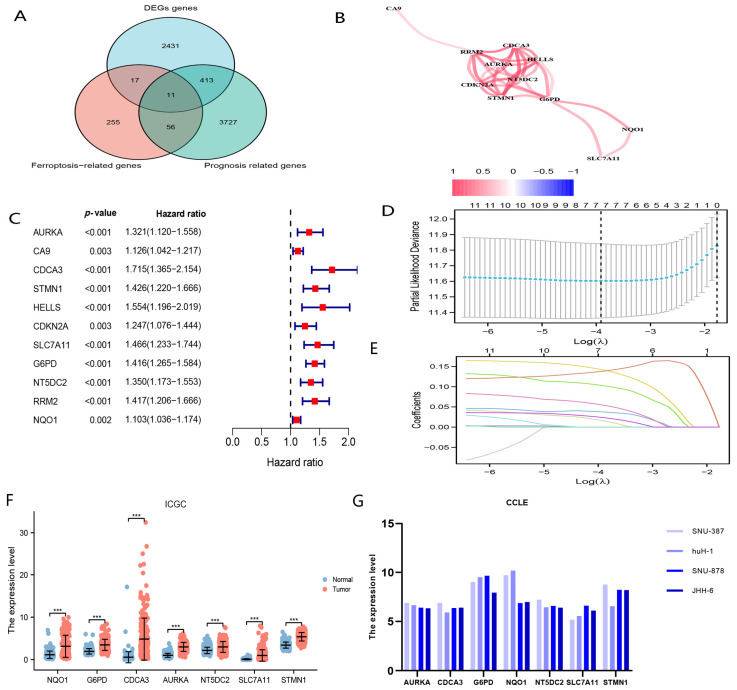
The procession before constructing nFRGs. (**A**) Screening candidate genes. (**B**) The correlation network of candidate genes. (**C**) Univariate Cox analyses of candidate genes. (**D**) LASSO coefficient profiles. (**E**) Candidate ferroptosis-related genes were filtered by the LASSO algorithm. (**F**) Verification of the expression level of genes consisting of nFRGs in ICGC. (**G**) Verification of the expression level of genes consisting of nFRGs in CCLE. (*** representing *p* < 0.001).

We obtained 2872 differentially upregulated genes, 4207 genes associated with OS and 339 ferroptosis-associated genes. The genes of these three groups were intersected to obtain 11 candidate genes (Figure 2A,C); the correlation of these 11 genes is illustrated in Figure 2B. Moreover, these 11 candidate genes were entered into the LASSO algorithm to obtain seven genes comprising the nFRGs (AURKA, CDCA3, STMN1, SLC7A11, G6PD, NT5DC2 and NQO1) (Figure 2D,E). We further confirmed the upregulated expression of these seven genes in HCC tissues in the ICGC and CCLE databases (Figure 2F,G).

nFRGs risk score = [AURKA × (0.01584)] + [CDCA3 × (0.03753)] + [STMN1 × (0.10271)] + [SLC7A11 × (0.1415856)] + [G6PD × (0.13851)]] + [NT5DC2 × (0.05450)] + [NQO1 × (0.02363)]

**Figure 3:**
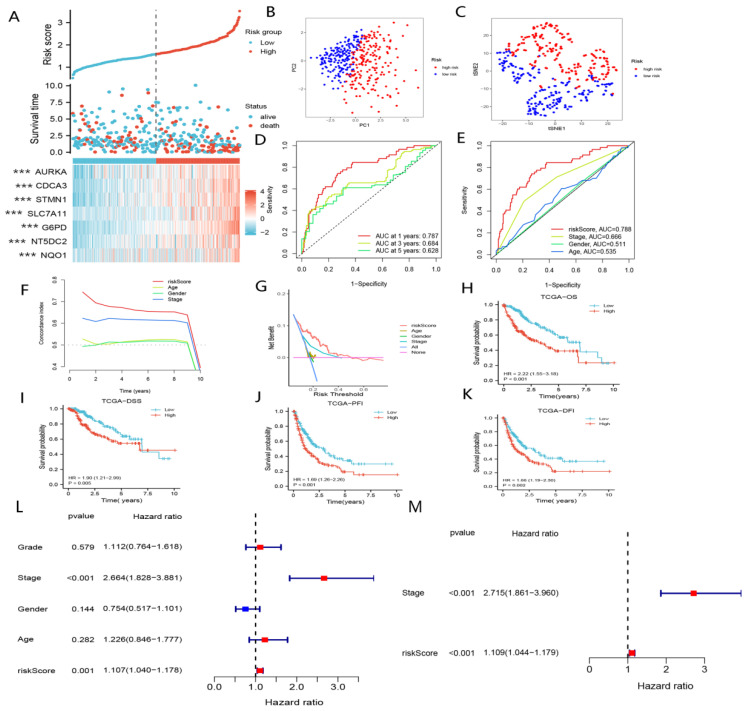
Construction of nFRGs in TCGA. (**A**) Risk score distribution map. (**B**) PCA plot. (**C**) t-NSE plot. (**D**) Time ROC curve of nFRGs. (**E**) ROC curve of nFRGs and other indicators. (**F**) C-index curve of risk score and other indicators. (**G**) DCA curve of risk score and other indicators. (**H**) KM curves of OS. (**I**) KM curves of DSS. (**J**) KM curves of DFI. (**K**) KM curves of PFI. (**L**) Univariate cox regression analysis of risk score and other indicators. (**M**) Multivariate cox regression analysis of risk score and TNM stage. (*** representing *p* < 0.001).

The 368 patients with HCC in the TCGA cohort were categorized into high-risk and low-risk groups based on the median risk scores. We discovered that patients with elevated risk scores suffered from shorter overall survival and higher mortality (Figure 3A,H–K). Patients in the high-risk group showed worse prognoses compared to those in the low-risk group (Figure 3H–K). In addition, PCA and t-NSE indicated a significant clustering of HCC patients in the low- and high-risk groups (Figure 3B,C). As shown in Figure 3D, nFRGs demonstrated excellent predictive performances with respect to prognoses in HCC patients. Moreover, nFRGs may be more favorable in assessing OS compared with TNM stage, age and gender (Figure 3E–G). Furthermore, nFRGs was identified as an independent risk factor for prognoses, according to Figure 3L,M.

**Figure 4:**
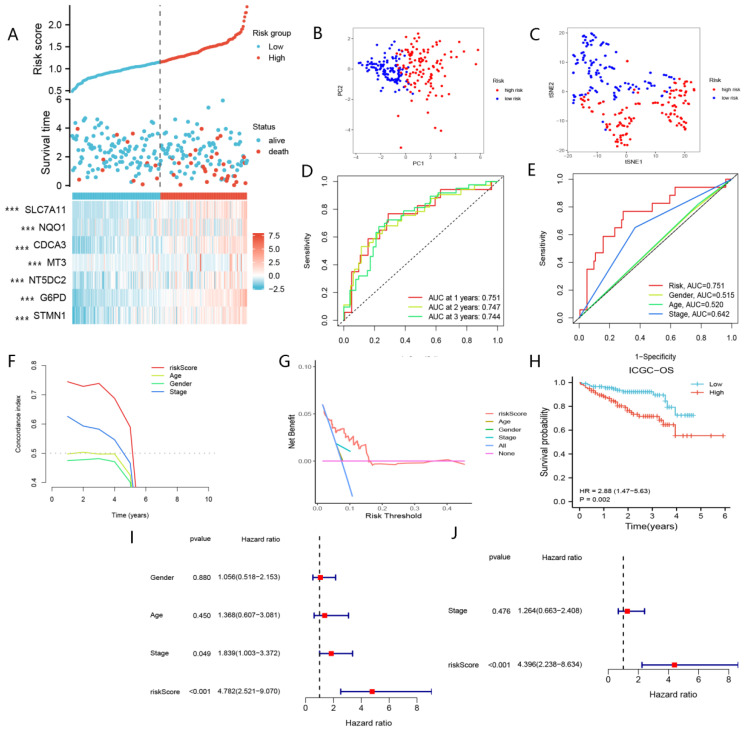
Validation of nFRGs in ICGC. (**A**) Risk score distribution map. (**B**) PCA plot. (**C**) t-NSE plot. (**D**) Time ROC curve of nFRGs. (**E**) ROC curve of nFRGs and other indicators. (**F**) C-index curve of nFRGs and other indicators. (**G**) DCA curve of nFRGs and other indicators. (**H**) KM curve of OS. (**I**) Univariate Cox analysis of risk score, gender, stage and age. (**J**) Multivariate Cox analysis of risk score and stage. (*** representing *p* < 0.001).

Risk scores were calculated for the 232 patients in the ICGC cohort using the same formula as in the TCGA cohort. The results for the ICGC cohort were generally consistent with those for the TCGA cohort (Figure 4A–J). Specifically, patients in the high-risk group presented with lower survival rates and shorter survival times. These findings illustrate the excellent performances of nFRGs in predicting the prognosis of HCC patients.

#### 4.1.2. Correlation between nFRGs and Clinicopathological Features

**Figure 5:**
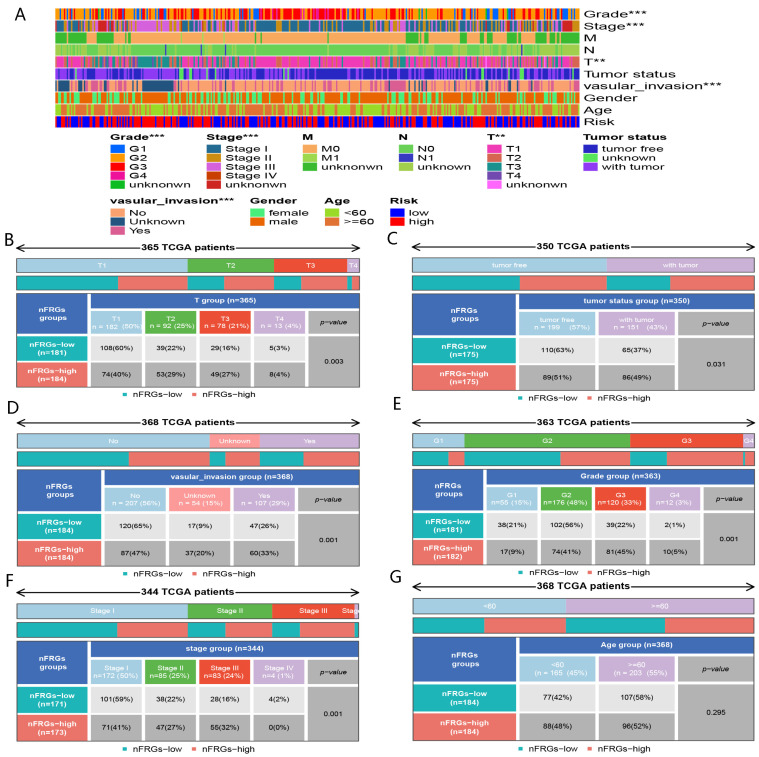
Correlation between nFRGs and clinical characteristics. (**A**) Correlation heat map. (**B**) Comparison of T1, T2, T3, and T4 in high- and low-risk groups. (**C**) Comparison of tumor-free and with tumor in the high- and low-risk groups. (**D**) Comparison of tumor-free and with tumor in the high- and low-risk groups. (**E**) Comparison of vascular invasion in the high- and low-risk groups. (**F**) Comparison of G1, G2, G3, and G4 in the high- and low-risk groups. (**G**) Comparison of stage I, and stage II, as well as stage III, and stage IV in the high- and low-risk groups. (**, *** representing *p* < 0.01 and *p* < 0.001, respectively).

Increased risk scores were observed for patients with advanced TNM stage, T stage, grade and vascular invasion status (Figure 5A–G), indicating that elevated risk scores could predict worse prognosis for HCC patients. These results reaffirm that nFRGs exhibited excellent performance in predicting the prognosis of HCC.

#### 4.1.3. Comparison of nFRGs with Other Gene Signatures

**Figure 6:**
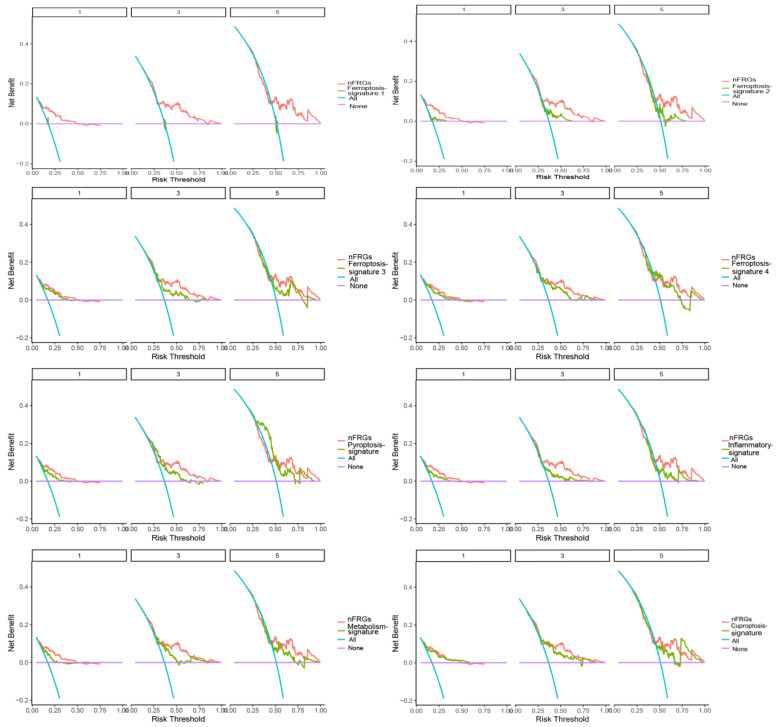
Comparison of nFRGs and other promising gene signatures.

As shown in Figure 6, nFRGs performed better in predicting the prognosis of patients with HCC compared to promising gene signatures involved in ferroptosis, cuproptosis, pyroptosis, inflammatory response and metabolism gene signatures [19,20,21,22,31,32,33,34]. These findings further demonstrate the strong potential to assess the prognoses of HCC patients.

#### 4.1.4. Development of nFRGs-Based Nomogram

**Figure 7:**
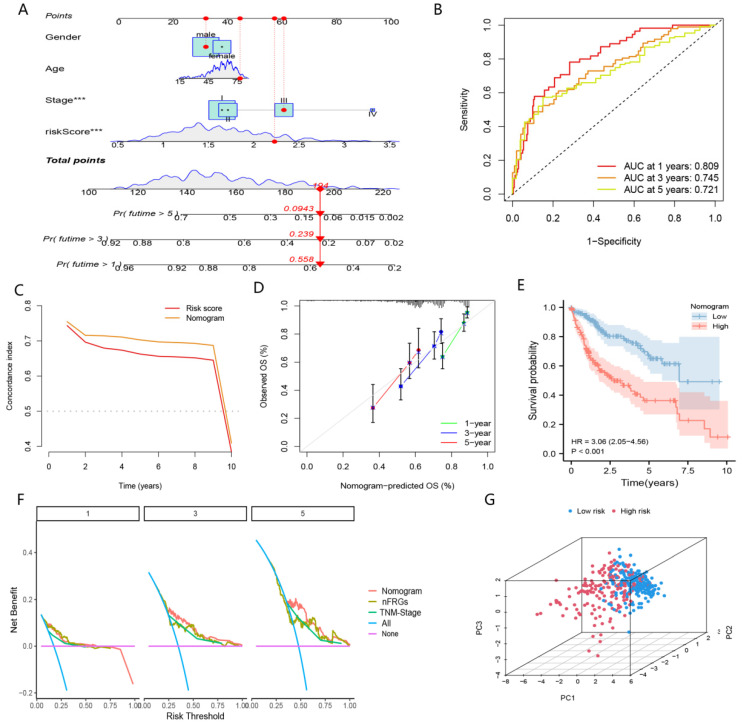
Nomogram based on nFRGs and other indicators. (**A**) Nomogram. (**B**) Time ROC curve of the nomogram. (**C**) C-index curve of the nomogram and risk score. (**D**) Calibration curve of the nomogram. (**E**) KM curve of nFRGs. (**F**) DCA curve of the nomogram, risk score and TNM stage. (**G**) PCA plot. (*** representing *p* < 0.001).

To better implement nFRGs in clinical practice, we developed a nomogram based on nFRGs and TNM stage in the TCGA cohort (Figure 7A). The calibration curve showed that the predicted survival at 1, 3 and 5 years was highly consistent with the actual survival (Figure 7D). As shown in Figure 7B, the AUC values at 1, 3 and 5 years were 0.809, 0.745 and 0.721, respectively. Furthermore, the C-index and DCA curves suggested that nomograms were superior to the TNM stage and risk scores in predicting the prognosis of patients with HCC (Figure 7C,F). Patients were stratified into high- and low-risk groups according to the median of the total nomogram scores. We observed a significant clustering of patients in the high- and low-risk groups (Figure 7G). Patients in the high-risk group showed worse prognoses than those in the low-risk group (Figure 7E). These results suggested that the nomogram based on nFRGs achieved excellent performances in predicting the prognosis of HCC patients, making it worthy of clinical promotion.

### 4.2. Stemness Index Analyses

**Figure 8:**
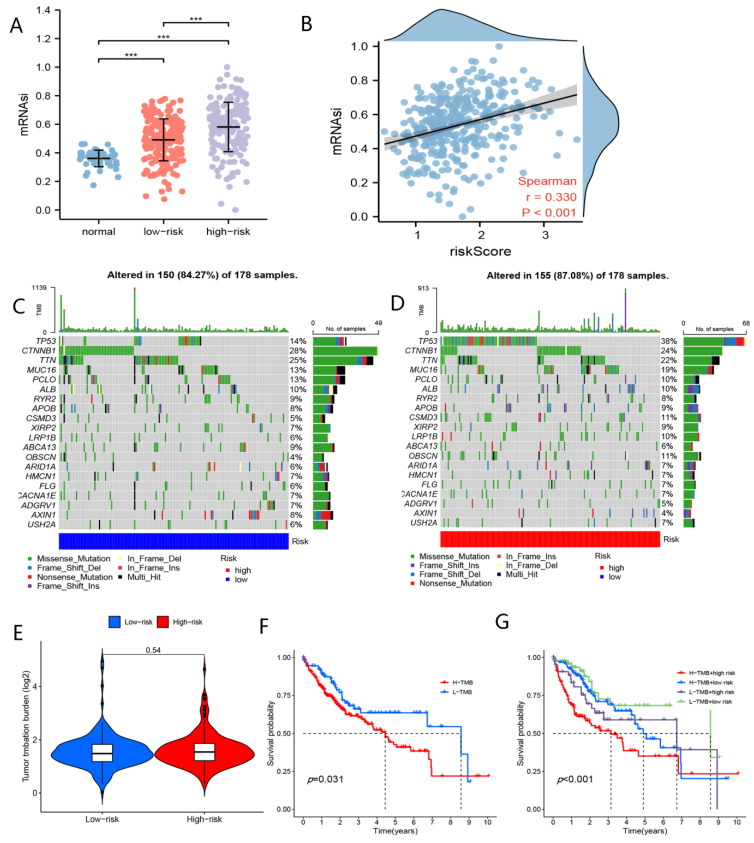
Stemness index and somatic mutation analyses. (**A**) Comparison of mRNAsi in the low- and high-risk groups. (**B**) Correlation between risk score and mRNAsi. (**C**) Waterfall plot for the top 20 mutated genes in the high-risk group. (**D**) Waterfall plot for the top 20 mutated genes in the high-risk group. (**E**) Comparison of TMB in the high- and low-risk groups. (**F**) KM curve of TMB. (**G**) KM curve of TMB + nFRGs. (*** representing *p* < 0.001).

As showed in Figure 8A,B, the mRNAi was greater in the high-risk group than in the low-risk group, and the risk scores were positively correlated with mRNAsi, which could explain the worse prognoses of patients in the high-risk group from the perspective of mRNAsi.

### 4.3. Somatic Mutation Analyses

Waterfall plots show the top 20 mutated genes in patients in the high- and low-risk groups (Figure 8C,D). As shown in Figure 8C,D, CTNNB1 mutations were most frequently observed in the high-risk group, and TP53 changes most commonly occurred in the low-risk group. TMB did not vary between the low- and high-risk groups (Figure 8E). However, there were marked differences in survival rates between patients with high TMB and those with low TMB (Figure 8F). Patients in the low-risk + high-TMB group had higher overall survival rates (*p* < 0.05) (Figure 8G).

### 4.4. Molecular Function Analyses

**Figure 9:**
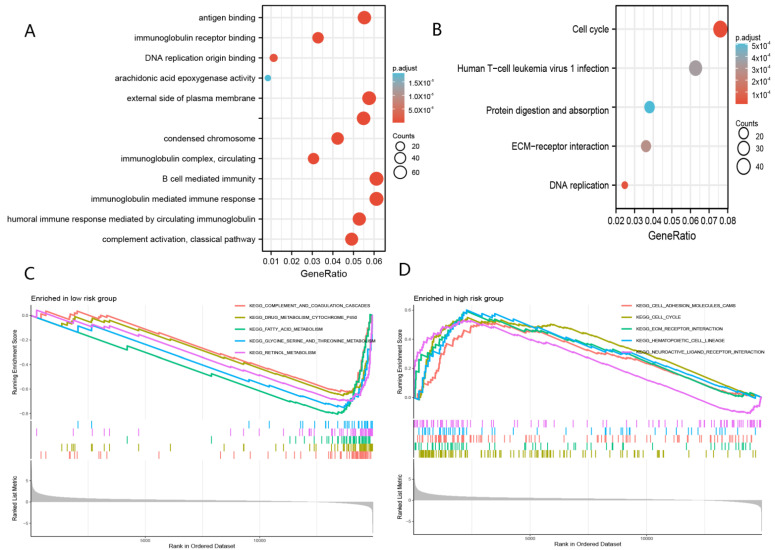
Molecular function analyses. (**A**) Go analysis of DEGs. (**B**) KEGG analysis of DEGs. (**C**) GSEA analysis in the high-risk group. (**D**) GSEA analysis in the low-risk group.

The results of GO analysis indicate that the molecular functions of DEGs were mainly enriched in B-cell-mediated immunity, immunoglobulin-mediated immune response, and immunoglobulin complex (Figure 9A). On the other hand, KEGG analysis showed that the molecular functions of DEGs were clustered in the cell cycle (Figure 9B). In addition, GSEA analysis revealed that many cancer metastatic pathways were significantly clustered in the high-risk group, including cell adhesion molecules (CAMs), cell cycle and ECM receptor interaction (Figure 9C). Interestingly, neuroactive ligand receptor interactions and hematopoietic lineage were also significantly clustered in the high-risk group. Furthermore, some metabolic pathways were enriched in the low-risk group, such as drug metabolism cytochrome P450, fatty acid metabolism, glycine serine, threonine metabolism, and retinol metabolism (Figure 9D).

### 4.5. The Role of nFRGs in Predicting Responses to Immunotherapy

**Figure 10:**
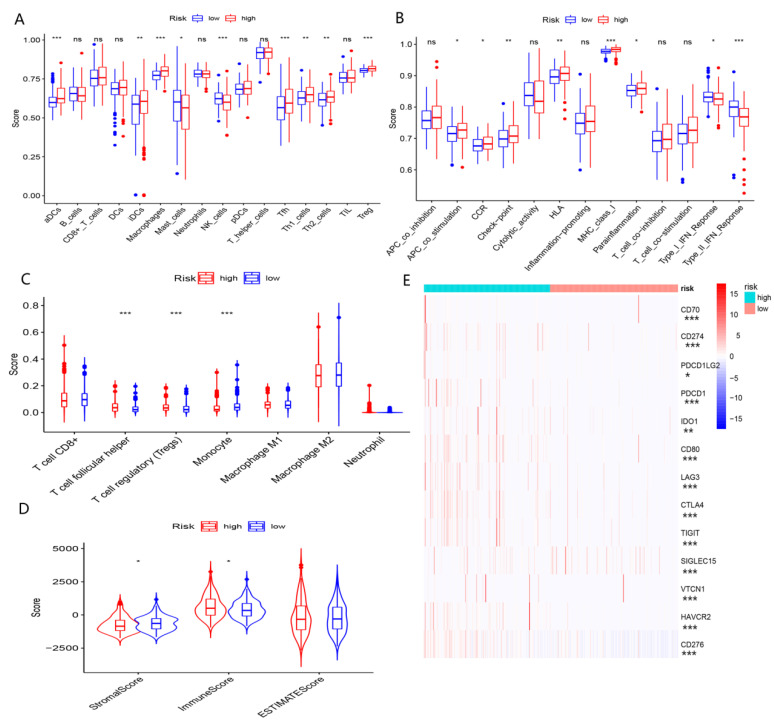
Immune profile analyses. (**A**) Comparison of immune cell infiltration between high- and low-risk groups using the ssGSEA algorithm. (**B**) Comparison of immune function between the high- and low-risk groups using the ssGSEA algorithm. (**C**) Comparison of immune cell infiltration between the high- and low-risk groups using the CIBERSORT algorithm. (**D**) Comparison of components in TME between the high- and low-risk groups. (**E**) Comparison of immune checkpoint in the high- and low-risk groups. (*, **, ***, and ns representing *p* < 0.05, *p* < 0.01, *p* < 0.001, and not statistically significant, respectively).

Our results demonstrated that enhanced “immune scores” were yielded in the high-risk group, whereas elevated “stromal scores” were observed in the low-risk group (Figure 10D). However, there appeared to be no significant difference between these two groups in terms of “ESTIMATE score” (Figure 10D). These findings suggested that the immune status was distinct between the high-risk and low-risk groups. As shown in Figure 10A,C, the low-risk group exhibited a significantly higher proportion of macrophages and NK cells, whereas the high-risk group had a higher abundance of Th2, Treg and Tfh cells. With respect to immune function, type-I interferon response and type- II interferon response were more stronger in the low-risk group of patients than the high-risk group (Figure 10B). More importantly, the expressions of common immune checkpoints were upregulated in patients in the high-risk group (Figure 10B,E).

**Figure 11:**
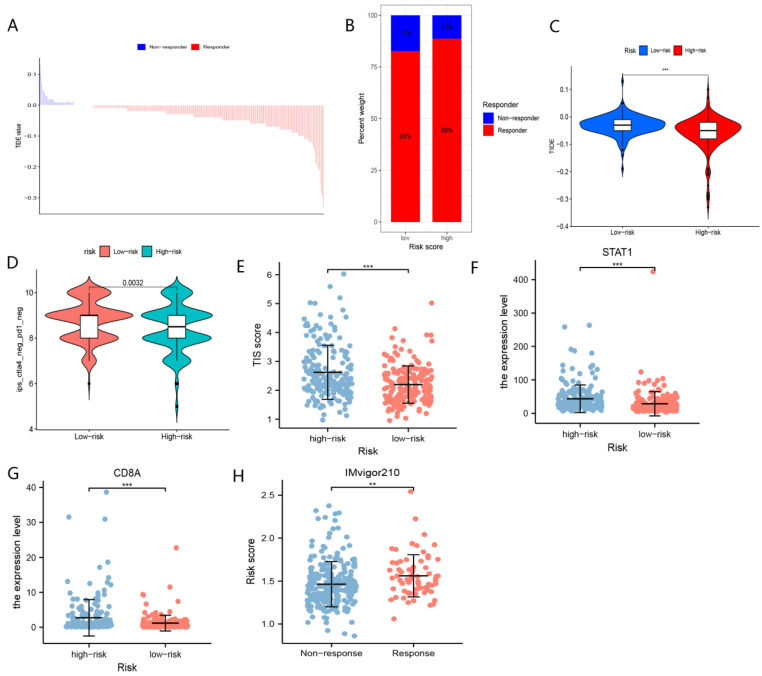
The role of nFRGs in predicting responses to immunotherapy. (**A**) The distribution of TIDE scores in responders and non-responders. (**B**) The proportion of responders and non-responders in the high- and low- risk groups. (**C**) Comparison of TIDE scores in high- and low-risk groups. (**D**) Comparison of IPS scores in high- and low-risk groups. (**E**) Comparison of TIS in the high- and low-risk groups. (**F**) Comparison of expression of STAT1 in the high- and low-risk groups. (**G**) Comparison of expression of CD8A in the high- and low-risk groups. (**H**) Comparison of risk scores in the responders and non-responders to immunotherapy. (**, *** representing *p* < 0.01 and *p* < 0.001, respectively).

As shown in Figure 11A, patients with lower TIDE scores may be more likely to benefit from immunotherapy. Patients in the high-risk group had lower TIDE scores than those in the low-risk group (Figure 11B,C), implying that patients in the high-risk group may be more likely to achieve a satisfactory outcome with immunological therapy. IPS scores verified that patients in the low-risk group were not sensitive to immunotherapy with anti-PDL1 or CTLA4 (Figure 11D).

Upregulated expression of CD8A and STAT1 has been demonstrated to predict the response of immunological therapy. As expected, elevated CD8A and STAT1 expressions were concentrated in the high-risk group of patients (Figure 11F,G).

It has been reported that TIS could be applied to assess the response to immunotherapy. Our study results revealed that patients in the high-risk group showed significantly higher TIS scores than those in the low-risk group (Figure 11E). Notably, enhanced risk scores yielded in this group of responders to immunotherapy in the IMvigor210 cohort (Figure 11H).

### 4.6. The Role of nFRGs in Predicting the Response to TACE

**Figure 12:**
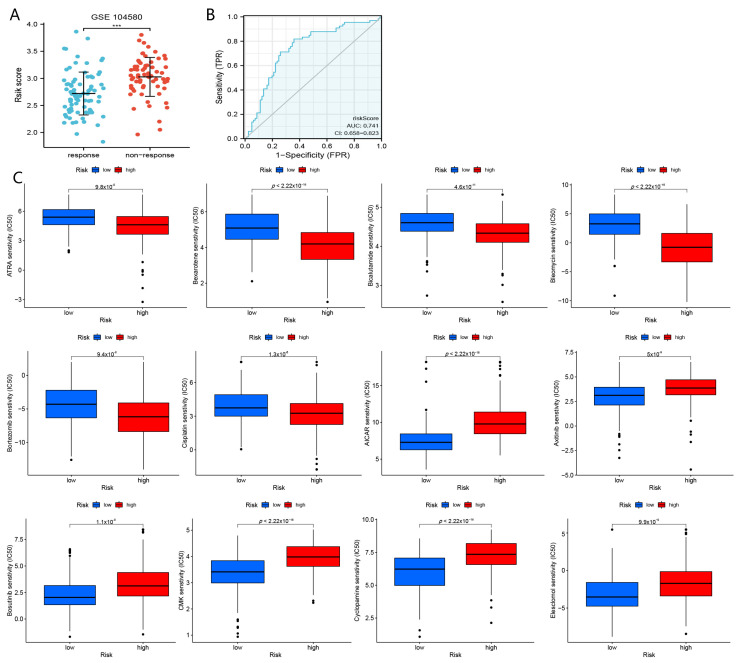
Analyses of response to TACE and drug sensitivity. (**A**) Comparison of risk scores in responders and non-responders to immunotherapy. (**B**) ROC curve of nFRGs evaluating the response to TACE. (**C**) Screening of sensitive drugs for the high- and low-risk groups. (*** representing *p* < 0.001).

As shown in Figure 12A, lower risk scores were observed in e patients who responded to TACE treatment (*p* < 0.01). In addition, the AUC of the risk score in assessing the response to TACE was 0.741 (Figure 12B). These results preliminarily clarified that nFRGs might be applied as a novel biomarker to assess the efficacy of TACE therapy in patients with HCC.

### 4.7. The Role of nFRGs in Drug Sensitivity

As shown in Figure 12C, we screened a total of nine drugs, of which “bleomycin”, “bortezomib”, “bicalutamide”, “ATRA” and “cisplatin” were determined to be more suitable for patients in the high-risk group, whereas “cyclopamine”, “AICAR”, “axitinib” and “CMK” may be more beneficial for patients in the low-risk group.

## 5. Discussion

In this study, nFRGs was created by applying the data from the TCGA cohort and validated in the ICGC cohort. Our results suggested that patients in the high-risk group had shorter survival times and higher mortality rate. nFRGs was superior to other promising gene signatures in predicting prognoses. Moreover, we discovered that the molecular functions, somatic mutations and mRNAsi differed between the high- and low-risk groups. In addition, patients in the high-risk group may be more susceptible to immunotherapy, whereas patients in the low-risk group may benefit more from TACE therapy. Furthermore, in this study, we selected sensitive drugs from more than 300 anticancer drugs for patients in the high- and low-risk groups. These results suggested that nFRGs may have considerable potential to predict prognosis, immunotherapy response, TACE efficacy and drug sensitivity in patients with HCC.

Several studies have demonstrated that pathways related to ferroptosis are involved in the progression of HCC [10,11,12]. Cancer cells may acquire resistance to ferroptosis by altering the expression of the genes [35]. We created nFRGs consisting of seven ferroptosis-related genes, including CDCA3, NQO1, STMN1, AURKA, G6PD, NT5DC2, and SLC7A11. CDCA3 has been demonstrated to be among the potential carcinogenic factors for HCC [36]. NQO1 promotes the invasion of HCC by amplifying the ERK-NRF2 signaling pathway [37]. In addition, STMN1 exacerbates HCC by triggering the hepatocyte growth factor (HGF)/MET signaling pathway [38]. It has been reported that knockdown of AURKA markedly inhibits the colony formation and migration ability of HCC cells [39]. Emerging evidence indicates that G6PD inhibits ferroptosis in HCC cells through POR, and G6PD depletion suppresses the growth and metastasis of HCC cells via upregulation of POR [40]. Furthermore, overexpression of NT5DC2 was reported to promote the proliferation of HCC cells in vitro and to facilitate tumor growth in vivo [41]. A growing body of evidence suggests that SLC711 facilitates proliferation of HCC cells and contributes to tumor advancement by inhibiting ferroptosis [10,42,43].

Using data from the TCGA and ICGC databases, we demonstrated that nFRGs achieved excellent performance in predicting the prognoses of HCC patients. In particular, patients in the high-risk group showed shorter survival times and increased mortality. nFRGs was also identified as an independent risk factor for the prognoses of patients with HCC. Notably, compared with other genes signatures, nFRGs might be more advantageous for prediction of prognoses in patients with HCC. We found that the predictive performance of the nomogram was greater than that of risk scores and TNM staging, and nomogram may be more suitable for clinical application. Taken together, our findings suggested that nFRGs excelled in assessing the prognoses of HCC patients.

Furthermore, we explored the factors responsible for the relatively worse prognosis in the high-risk group based on the findings obtained in this study.

The profiles of genetic mutations may contribute to the distinct prognoses of two subgroups. In this study, the frequency of mutations seemed to be higher in the high-risk group. CTNNB1 and TP53 mutations were most frequently observed in high- and low-risk groups, respectively. It has been reported that enhanced genomic instability is closely associated with relatively poorer prognoses of patients with malignant tumors [44]. In addition, HCC tissues with CTNNB1 mutations are generally characterized by better differentiation and lower grade [45]. In contrast, HCC tissues with TP53 mutations are characteristic of hypodifferentiation, vascular invasion and angiogenesis [45]. Consequently, the genetic mutation profile may contribute to the diverse prognoses of patients in the high- and low-risk groups.

Distinct molecular functions may be responsible for this phenomenon as well. In our study, many cancer metastatic pathways were found to be markedly aggregated in the high-risk group, such as cell adhesion molecules (CAMs), cell cycle and ECM receptor interactions. In addition, metabolism-related pathways were enriched in the low-risk group in terms of drug metabolism cytochrome P450, fatty acid metabolism, glycine serine, threonine metabolism, and retinol metabolism. Thus, distinct molecular functions may lead to varying outcomes between the high- and low-risk groups.

Furthermore, elevated mRNAsi yielded in the high-risk group. Higher mRNAsi has been reported to be positively correlated with the dedifferentiation and aggressiveness of tumor cells [46], which may help to account for the poorer prognoses of patients in the high-risk group.

As molecular functions of DEGs between subgroups were enriched in immune-related pathways, we hypothesized that immune features may result in the diverse outcomes between these subgroups. Our results revealed that the low-risk group presented with a higher proportion of macrophages and NK cells, in combination with enhanced type-I IFN responses and type-II IFN responses. Nevertheless, the high-risk group showed a greater abundance of Th2 cells and Treg cells, as well as upregulated expression of immune checkpoints. A substantial body of evidence suggests that Th2 cells and Treg cells may promote immune escape from malignant tumors, including HCC [47]. In contrast, NK cells exert a powerful antitumor effect. Recent evidence demonstrated that elevated expression of immune checkpoints gives rise to an immunosuppressed state in the tumor microenvironment. According to our study, there was a noteworthy state of immunosuppression in the high-risk group of patients, suggesting that the worse prognoses of HCC patients in high-risk group may correlate with immune profiles.

Based on the nFRGs, more precise and personalized treatment may be administered to patients with HCC in the high- and low-risk groups.

Our study suggested that patients in the high-risk group might achieve satisfactory outcomes from immunotherapy. In this study, the enhanced expression of CD274, CD8A, and STAT1 was clustered in high-risk group, and a wealth of studies have indicated that patients with upregulated expression of PD-L1 (CD274), CD8A, and STAT1 may be more likely to benefit from immunotherapy. In addition, the TIDE, IPS, and TIS scores demonstrated that patients in the high-risk group may be more sensitive to immunotherapy, whereas patients in the low-risk group were relatively impervious to immunologic therapy. More importantly, in the IMvigor210 cohort, patients who responded to immunotherapy showed considerably higher risk scores than those who did not respond to immunotherapy. In addition, tumor tissues with CTNNB1 mutation are impervious to immunotherapy [48], whereas patients with TP53 mutation may be more likely to respond to immunotherapy [49]. These results demonstrated that patients in the high-risk group may be more sensitive to immunotherapy from diverse perspectives.

TACE therapy remains a potential option for patients with bulky HCC that cannot be surgically resected. However, clinicians always have trouble in selecting more suitable patients with HCC for TACE treatment. Our study suggested that patients in the low-risk group may be more susceptible to TACE treatment. The AUC of risk score to predict TACE response was determined to be 0.741, indicating that nFRGs may be accessible as a novel biomarker for assessing the efficacy of TACE.

Chemotherapy and targeted therapy remain pivotal for the treatment of HCC. Nonetheless, it is challenging to select patient-sensitive drugs from more than 300 drugs. In this study, we discovered that patients with HCC in the high-risk group may be more susceptible to “bleomycin”, “bortezomib”, “bicalutamide”, “ATRA” and “cisplatin”, whereas HCC patients in the low-risk group may be more sensitive to “cyclopamine”, “ecard”, “axitinib” and “CMK”. Notably, screening for sensitive drugs for diverse subgroups might guide clinicians in personalizing treatment of HCC patients.

In this study, we systematically explored the potential of nFRGs to predict prognoses, responses to immunotherapy, efficacy of TACE therapy and drug sensitivity in patients with HCC, with promising clinical implications. More importantly, compared with other published gene signatures, nFRGs performed more favorably in predicting the prognosis of HCC. And we further obtained the hub gene (SLC7A11) from these seven genes using multivariate Cox regression analysis and the analysis of SLC7A11 was detailed in the Figures S1–S4. However, our study is subject to several limitations. First, similarly to other studies [19,20,21,22,31,34], we applied relevant data from public databases, and further basic experiments will be required in the future. In addition, we were unable to classify HCC patients based on etiology, owing to incomplete information from these datasets. In conclusion, nFRGs achieved excellent performances in predicting the prognosis of HCC patients. Notably, in comparison with other genes, nFRGs was more advantageous in assessing OS of patients with HCC. Of note, nFRGs could be applied as a novel biomarker to assess immunotherapy response, TACE efficacy, and drug sensitivity. Thus, nFRGs might play a crucial role in guiding clinicians in making personalized treatment decisions for patients with HCC.

## 6. Disclosure

TCGA, GEO, CCLE, IMvigor210 and ICGC belong to public databases. The patients involved in these databases have provided ethical approval. Users can download relevant data for free for research and publish relevant articles. Our study is based on open-source data, so there are no ethical issues or other conflicts of interest.

## Data Availability

The data used to support the findings of this study are available from the corresponding authors upon request.

## Supplementary Materials

The following supporting information can be downloaded at: https://www.mdpi.com/article/10.3390/curroncol29100550/s1, Figure S1: KM curves of SLC7A11 and Cox regression analysis; Figure S2: Nomgram containing FRGS and stage; Figure S3: Immune profile analysis; Figure S4: Target drugs of SLC7A11.

## Abbreviations

nFRGs	Novel ferroptosis-related genes signature
HCC	Hepatocellular carcinoma
DEGs	Differentially expressed genes
LASSO	Least absolute shrinkage and selection operator
TCGA	Cancer Genome Atlas
TMB	Tumor mutation burden
OS	Overall survival
TME	Tumor microenvironment
