# Supplementary material for: A Novel Ferroptosis-Related Signature for Prediction of Prognosis, Immune Profiles and Drug Sensitivity in Hepatocellular Carcinoma Patients
Source: Curr Oncol. 2022 Sep 27;29(10):6992–7011. doi: 10.3390/curroncol29100550 (PMC9601138; doi:10.3390/curroncol29100550)
Supplement: Supplementary file 1 [file curroncol-29-00550-s001.zip › curroncol-1870399-supplementary.pdf]

NQO1	1.102 (1.039-1.168)	**0.001**	1.040 (0.973-1.110)	0.250

Supplementary description

We cross-validated that over expression of SLC7A11 was associated with poor prognosis of HCC using TCGA and ICGC databases, and also demonstrated that high expression of SLC7A11 was an independent risk factor for OS in HCC (Figure S1). Moreover, we constructed a nomogram model based on patholoIgical stage and SLC7A11 expression level, and the calibration curve and DCA curve demonstrated the reliability of its predictive ability (Figure S2). We found that the nomogarm model incorporating SLC7A11 was similar to the nomogram model including nFRGs in predicting 1-year survival rate of HCC. Furthermore, we also explore the correlation between the expression of SLC7A11 and immune features (Figure S3).In the GDSC database, we also screened the target drugs for SLC7A11 (Figure S4). Thus we can develop personalized treatment for HCC patients based on the expression of SLC7A11 when we did not detect the expression of the other six genes in a timely manner.

Figure S1

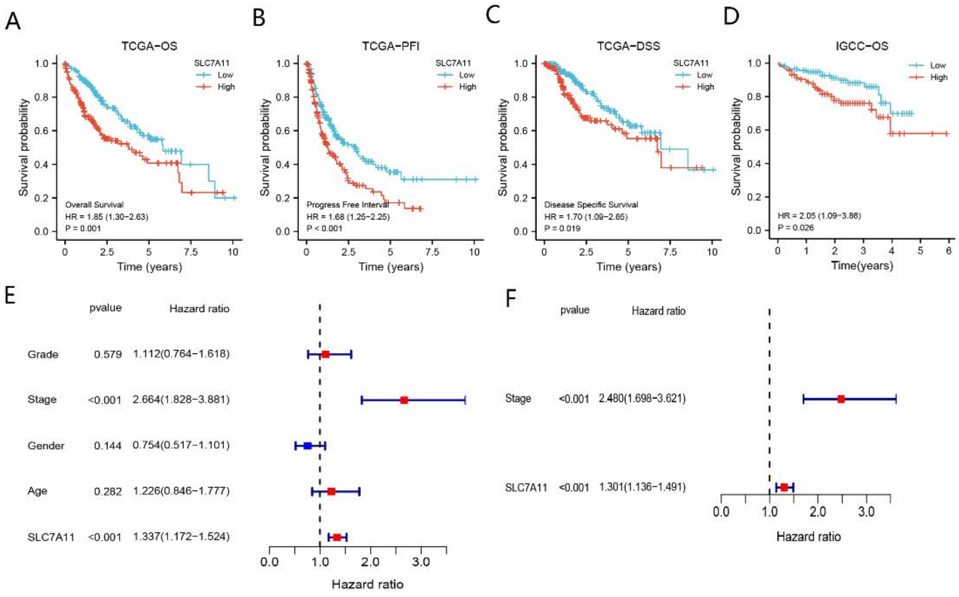

Figure s2

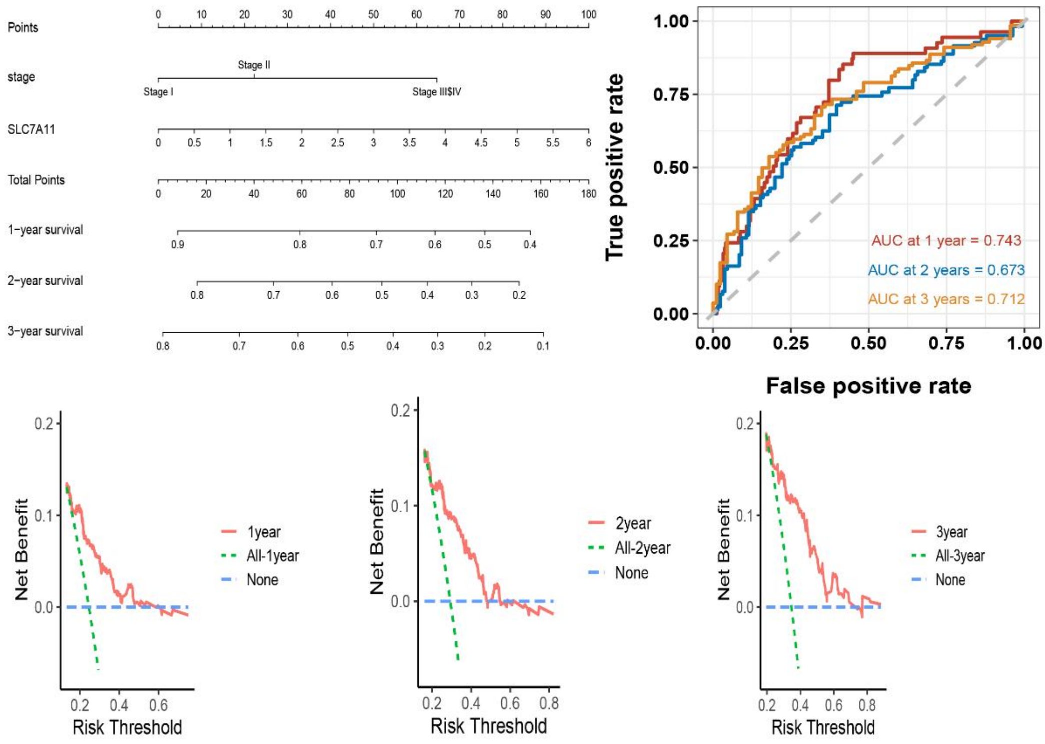

FigureS3

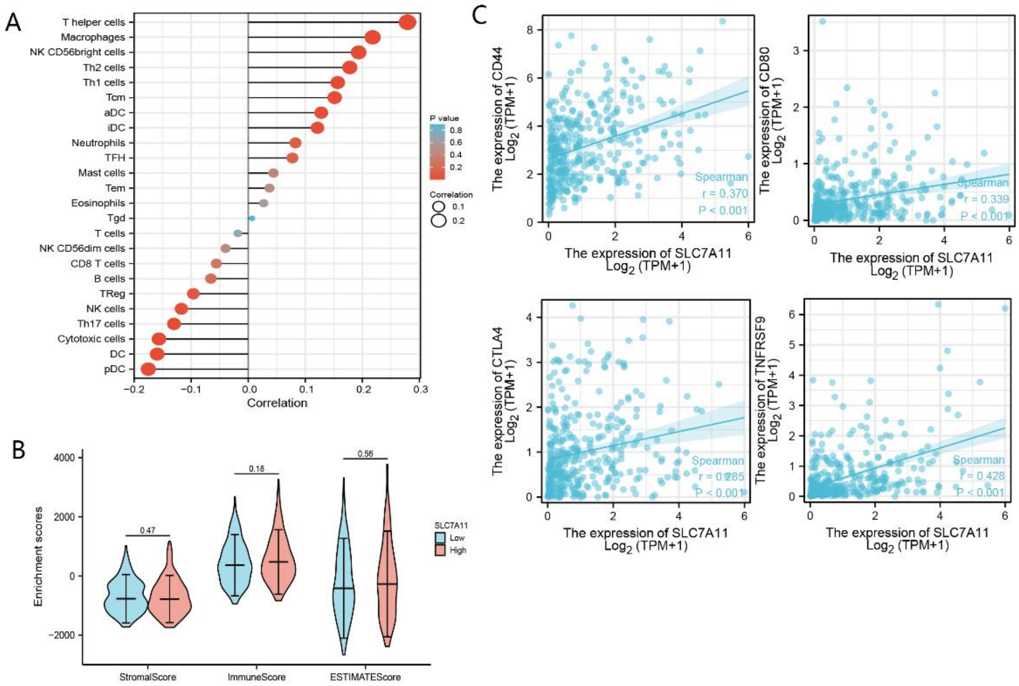

Figure S4

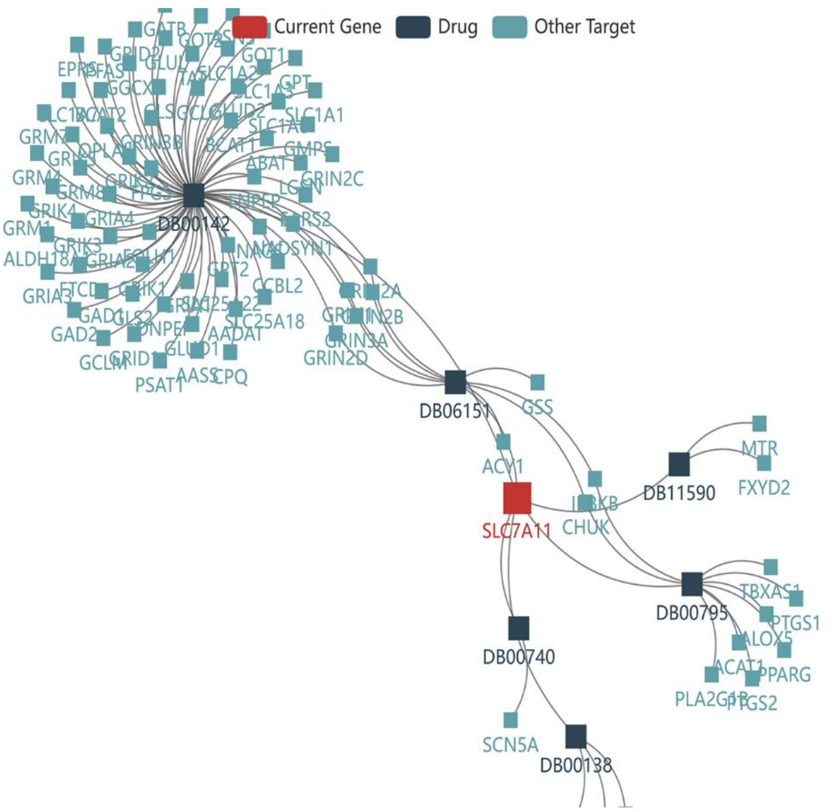

Figure S1: KM curves of SLC7A11 and Cox regression analysis (A)TCGA- OS. (B)TCGA-PFI. (C)TCGA-DSS. (D)ICGC-OS. (E) Univariate cox regression analysis in TCGA.(F) Multivariate cox regression analysis in TCGA

Figure S2: Nomgram containing FRGS and stage (A) Nomgram. (B)ROC curve. (C) DCA curve of 1 year. (D) DCA curve of 2 year. (E) DCA curve of 3 year.

Figure S3: Immune profile analysis. (A) the correlation between SLC7A11 and Immune cell infiltration. (B) Estimate analysis. (C) the correlation between SLC7A11 and CTLA4, CD80, CD44, TNFRSF9.

Figure S4: Target drugs of SLC7A11.
